# Depression, Anxiety, and Psychological Resilience in Healthcare Workers during the Pandemic (COVID-19)

**DOI:** 10.3390/healthcare12191946

**Published:** 2024-09-28

**Authors:** Elif Yöyen, Tülay Güneri Barış, Fatih Bal

**Affiliations:** 1 Department of Psychology, Faculty of Humanities and Social Sciences, Sakarya University, Sakarya 54050, Turkey; 2 Department of Health Sciences, Institute of Business Administration, Sakarya University, Sakarya 54050, Turkey

**Keywords:** pandemic, anxiety, depression, psychological resilience, healthcare workers

## Abstract

Background/Objectives: The aim of this study is to examine the relationship between depression, anxiety, and psychological resilience variables with working conditions and various demographic variables in healthcare professionals who are actively involved in the pandemic process. Methods: This study included 1440 healthcare workers in different professions in two state hospitals accepted as pandemic hospitals. The research data were collected with the Sociodemographic Data Form, Hospital Anxiety and Depression Scale (HAD), and Psychological Resilience Scale (PRS). Independent samples *t*-test, one-way analysis of variance (ANOVA), and descriptive analyses were used to analyze the data. Results: As a result of the analyses, female participants had higher anxiety scores than male participants; healthcare professionals working as nurses, midwives, and health officers had higher anxiety scores than other healthcare professionals; those with less professional experience had higher anxiety scores than those with more professional experience; and those who had long hours of contact with COVID-19-positive patients (8 h or more per day) had higher anxiety scores than those with less contact. Single female healthcare workers reported higher depression symptoms than married female healthcare workers, those with children reported higher depression symptoms than those without children, and those with average professional experience (6–10 years) reported higher depression symptoms than those with more experience. Being single, having children, and having an average number of shifts (working at night) caused an increase in psychological resilience. Conclusions: The results of the study may contribute to the structuring of health policies to protect and support the mental health of healthcare workers in ongoing and future pandemic processes.

## 1. Introduction

Throughout history, the world has experienced many epidemics. If an epidemic spreads rapidly among people and is caused by a new virus, these epidemics are considered pandemics [1]. On 1 December 2019, the World Health Organization (WHO) China Country Office reported cases of pneumonia of unknown etiology in Wuhan, Hubei province, China [2]. In February 2020, the World Health Organization named this new type of virus “Coronavirus disease-2019 (COVID-19)” and the COVID-19 outbreak was declared as a pandemic after the disease spread rapidly worldwide and the number of cases and deaths increased [3].

The pandemic has seriously negatively affected the health and wellbeing of millions of people worldwide and caused a significant number of human casualties [4]. Studies show that during the pandemic period, people experienced various psychological problems such as depression [5], post-traumatic stress disorder [6], sleep and anxiety disorders [7], and burnout syndrome [8]. With the addition of factors such as stress, loneliness, and difficult economic conditions, findings have been presented that anxiety and concerns about COVID-19 lead to generalized anxiety disorders [9,10], occupational burnout, anxiety, and depression [11].

The COVID-19 pandemic, which started in late 2019 and gradually spread all over the world, has affected all people, but the most affected professional group is undoubtedly the frontline healthcare professionals. One of the first period studies on this subject was carried out in Italy. In this study, it was reported that 50% of healthcare workers had symptoms of post-traumatic stress disorder, 25% had symptoms of depression, and 20% had symptoms of anxiety [12]. In the same study, those who lost their colleagues due to COVID-19 reported more post-traumatic stress and depression symptoms. In a study conducted in Japan, it was found that more than 40% of nurses and more than 30% of radiology specialists and pharmacists working in the fight against COVID-19 showed the symptoms of burnout [13]. El-Hage and colleagues reported minimal depression in 62% of healthcare workers, mild depression in 21.5%, moderate depression in 13.5%, and severe depression in 3% [14]. In another study, it was determined that anxiety, depression, and PTSD syndromes were higher in healthcare workers who had direct contact with infected patients [15]. Similar results are noteworthy in studies conducted during previous epidemic/pandemic processes. After the 2003 SARS outbreak, high depression, anxiety, and post-traumatic stress disorder were reported one year after the outbreak in healthcare workers who actively took part in the fight against the epidemic [16]; alcohol use disorder, depression, PTSD, and burnout were reported in healthcare workers three years after the SARS-CoV-1 outbreak [17].

However, psychological resilience may be protective in terms of mental health during pandemics and other extraordinary times [18]. Psychological resilience, which generally refers to a process of success or adaptation [19], is seen as the adaptation process of the individual in the face of a vital stress (a trauma, a threat, a tragedy, etc.) [20]. In other words, it is defined as the power of the individual to recover himself/herself in difficult life experiences [21], and the ability of the individual to successfully overcome the changes or disasters that occur [22]. At first glance, psychological resilience is seen as a personality trait that reduces the negative effects of stress and supports adaptability [23]. In this respect, some studies focus on genetic characteristics and suggest that some people are born resilient [24]. However, studies have also revealed that psychological resilience is a personal trait that can be learnt [25]. Psychological resilience is a phenomenon that is perceived and realized in the face of the realities faced, learned, and involves a developmental process [26].

For psychological resilience to develop, a stressor must be encountered. Especially considering the intense stress periods of the pandemic process (fear of contracting the virus, fear of transmitting the virus to others, witnessing the infection and/or death of colleagues, long working hours, difficulties of working with protective equipment, efforts to balance work, family and social life, etc.), psychological resilience is an important concept for healthcare workers who encounter many stressors. In the literature, there are studies evaluating psychological resilience in terms of healthcare workers [27,28,29]. However, studies on psychological resilience in the COVID-19 process are not available in the country where the research was conducted.

There are also studies examining the relationship between working conditions, demographic variables, and the mental health of healthcare workers during the pandemic period. Studies of working conditions have reported that long working hours, night work in addition to day work (24 h continuous), and a high number of shifts (in a month) cause chronic insomnia, fatigue, errors, burnout, and poor concentration [30,31]. It has been reported that prolonged contact with COVID-19-positive patients places healthcare workers at high psychological and physiological risk for the disease, and that their susceptibility to infection increases as a result of weakening of the immune system due to long working hours and excessive fatigue [32,33]. Studies of demographic data have reported that anxiety and depression are more common in younger age groups and in women, that post-traumatic stress disorder is more common in primary care workers and general practitioners, and that severe insomnia is more common in nurses and healthcare assistants than in other professions [12]. However, there is no study that evaluates all the variables in this research together. The aim of this study is to examine the relationship between depression, anxiety, and psychological resilience factors in healthcare professionals who are actively working during the pandemic process and working conditions (how many hours they come into contact with COVID-19-positive patients during the daily shift, how many shifts they keep per month) and demographic variables (age, educational status, marital status, having children, profession, and years of professional experience).

The research questions created and tested in this context are as follows:

1. Is the level of anxiety in healthcare professionals who are actively working in the pandemic process related to the characteristics listed above?
2. Is the level of depression in healthcare professionals actively working in the pandemic process related to the characteristics listed above?
3. Is the level of psychological resilience in healthcare professionals actively working in the pandemic process related to the characteristics listed above?

## 2. Materials and Methods

### 2.1. Methods

For the research, permission was obtained from the Ethics Committee of Sakarya University Rectorate in Turkey on 13 January 2021 with protocol number 30/07. With the permission obtained, an application was made to the Provincial Health Directorate of Sakarya Governorship and the application was accepted with the study permit numbered E-18343338-434.99. Data were collected between February 2021 and December 2021 from two hospitals designated as pandemic hospitals for the study. This period is the period when a pandemic wave was experienced in the country. During this pandemic wave period, due to the measures taken by the Ministry of Health and the Ministry of Interior, some hospitals were declared as pandemic hospitals, the leaves of healthcare workers were cancelled and all healthcare workers were invited to work, and the rules of social restriction and isolation in the country were eased in the period of July–September 2021, but other periods were applied with all their restrictions (curfews, intercity and international travel restrictions, transition of all institutions to online work, etc.). In this context, the research data were provided by the researchers to the health workers. The researchers approached the managers of the hospitals with the permission documents mentioned above. The contact details of the healthcare workers were obtained from the managers, and the participants were contacted online. The data were delivered online by the researchers to the participants who volunteered to participate in the study and met the inclusion and exclusion criteria of the study. A total of three reminder emails were sent to participants at two-month intervals. A total of 1470 participants who responded to these calls were included in the study. The sample is a convenience sample. The exclusion criteria in the study were the absence of any psychiatric or physiological disease diagnosis (until the time of inclusion in the study), the absence of a regular medication, the absence of any psychological support currently or previously, and no previous experience of social disaster. These criteria were formed as questions in the Google questionnaire. Health professionals who did not meet the criteria were not allowed to fill in the other questions. The criteria for inclusion in the study were not working as an administrator/manager, and actively working in outpatient clinics, inpatient clinics, emergency, and intensive care units in pandemic hospitals. Informed consent was obtained from the participants before starting the study. In the analyses of the data obtained with the data collection tools, 30 participants were excluded from the dataset due to incomplete completion of the data. Analyses were carried out with the data of 1440 participants. SPSS 21.0 package programme was used to analyze the data.

In the research, 41.02% of the participants were male and 56.93% were female. The age distribution of the sample was 21.36% aged 25 years and under, and 23.74% aged 26–35. A total of 46.46% of the participants were single and 51.49% were married. The rate of individuals with children in the sample was 48.91%. When the educational status was analyzed, it was seen that 33.74% of the sample were university graduates. A total of 43.06% of the sample were working as midwife-nurse and health officer. A total of 29.11% of the participants had less than 5 years of professional experience and 26.05% had more than 16 years of professional experience. The rate of those working in shifts was 56.19%, while the rate of contact with COVID-19-positive patients was 52.04%. The results are presented in Table 1.

### 2.2. Data Collection Tools

#### 2.2.1. Sociodemographic and Working Conditions Questionnaire Form

It is a form that includes information on gender, age, educational status, marital status, having children, profession, working year/duration, number of shifts per month, and daily contact time with COVID-19 patients of the participants who were thought to be risk factors in being affected by the pandemic after the literature [12,30] was reviewed by the researchers.

#### 2.2.2. Hospital Anxiety and Depression Scale (HAD)

The HAD, a self-report scale developed by Zigmond and Snaith, aims to determine the risk of anxiety and depression in individuals and to measure the level and severity change. It takes 2–5 min to complete the scale. The fourteen-item HAD includes the Anxiety subscale (HAD-Scale-A) and Depression subscale (HAD-Scale-D), each consisting of 7 items. Each item is scored between 0–3 points by choosing from four options. The items are marked according to how the person has felt in the last week. Items 1, 3, 5, 6, 8, 10, 11, and 13 show gradually decreasing severity and are scored as 3, 2, 1, 0; items 2, 4, 7, 9, 12, and 14 are scored as 0, 1, 2, 3. For the Anxiety subscale, the scores of items 1, 3, 5, 7, 9, 11, and 13 are summed; for the Depression subscale, the scores of items 2, 4, 6, 8, 10, 12, and 14 are summed. By adding the subscale scores, 0–21 points can be obtained from each of the Depression and Anxiety subscales. For each subscale, it is stated that a score of 0–7 is a normal range, a score of 8–10 suggests the presence of a mood disorder, and a score of 11 and above indicates a possible mood disorder [34]. In this study, the scale reliability was found to be 0.86 for the anxiety subdimension, 0.87 for the depression subdimension, and 0.90 for the entire scale.

#### 2.2.3. Psychological Resilience Scale (PRS)

Psychological resilience, which functions as a source of resistance when faced with stressful life situations and is considered as a personality trait, is a self-report scale consisting of 21 items and three subdimensions that expresses that individuals are easily committed to the things they do in their lives, that they can control the situations they encounter in life, and that they evaluate changes in life as an opportunity to improve themselves. The scale developed by Işık is a five-point Likert scale in the form of “Strongly disagree”, “Disagree”, “Undecided”, “Agree”, and “Strongly agree”. Scoring of the items varies between 0 and 4 points. Items 2 and 15 are scored in the opposite direction. The increase in the scores obtained from the subdimensions of PBL and the overall scale indicates a high level of psychological resilience. The Cronbach alpha reliability coefficients for the subdimensions named as dedication, control, and challenge were determined between 0.62 and 0.74, respectively, and the Cronbach alpha reliability coefficient for the whole scale was determined as 0.76 [35]. In this study, the scale reliability of the subfactors for PBL were 0.85 for challenge, 0.81 for self-dedication, and 0.84 for control, respectively, and it was concluded that the factors were highly reliable. For the whole scale, it was measured as 0.90.

### 2.3. Data Analysis

Before analyzing the data, the responses of the participants with extreme values were removed from the dataset. Normality analyses were performed to test whether the data were suitable for the planned analyses. The normality analysis of the comparisons in the study was determined by kurtosis and skewness analyses [36]. In the study, multiple comparisons were determined by Tukey’s-b analysis. Total and subscale means, standard deviations, minimum and maximum scores of the scales used in the study, and Cronbach alpha values for reliability were calculated. In the analysis of the data, comparative difference tests were performed with *t*-test and Anova. Sociodemographic data were investigated with descriptive analyses. In descriptive analyses, mean and standard scores obtained from scales, and frequency and percentage values for demographic data were calculated. The independent groups *t*-test was used for two groups in the comparison analysis between groups, one-way analysis of variance for three or more groups, and descriptive analyses were used for the demographic information of the participants. Regression analysis was used to examine the effect of the independent variable on the dependent variable. All data were analyzed using SPSS software version 21.0, significance was tested at the *p* < 0.05 level, and other levels of significance were reported separately.

## 3. Results

The mean anxiety score was 11.03 (sd = 38.85), the mean depression score was 9.95 (sd = 0.35), the mean challenge score was 25.96 (sd = 1.12), the mean dedication score was 17.74 (sd = 0.49), and the mean control score was 10.16 (sd = 0.89). The normality test, which is the first stage of the analysis, was applied and the kurtosis and skewness values were checked. As the kurtosis and skewness values were between −2 and +2, it was decided that there was a normal distribution. The results are presented in Table 2.

The results of the independent sample *t*-test indicate that there were statistically significant differences in anxiety scores between genders (*p* = 0.000). The mean anxiety score for women was higher than that for men. Conversely, there were not statistically significant differences detected in depression (*p* = 0.099), challenge (*p* = 0.991), dedication (*p*-value = 0.284), and control factor (*p* = 0.167) scores contingent on gender. The results are presented in Table 3.

The results of the ANOVA test indicated a statistically significant difference in anxiety scores between age levels (*p* = 0.000). The Tukey-B results of multiple comparisons revealed that health workers in the 46–55 age group exhibited lower anxiety scores than those in other age groups. It was determined that there was a statistically significant correlation between age and depression (*p* = 0.047). The group comprising individuals aged 56 years and over exhibited a lower prevalence of depressive symptoms compared to the other age groups. Conversely, no significant difference was observed in the scores for the challenge factor (*p* = 0.912), dedication factor (*p* = 0.310), and control factor (*p* = 0.711). The results are presented in Table 4.

The results of the independent sample *t*-test indicate that there were statistically significant differences in anxiety scores based on gender and marital status (*p* = 0.000). The dedication scores of single males were higher than those of married males. Conversely, no statistically significant difference was identified in the depression (*p* = 0.426), challenge (*p* = 0.586), anxiety (*p* = 0.064), and control factor (*p* = 0.888) scores of marital status.

The independent sample *t*-test revealed a statistically significant difference in anxiety scores between gender and the female variable (*p* = 0.000). The scores for depression and challenge for female single individuals were higher than for those who were married. Conversely, no significant difference was identified in the anxiety (*p* = 0.141), dedication (*p* = 0.947), and control factor (*p* = 0.363) scores of female marital status. The results are presented in Table 5.

The results of the independent sample *t*-test indicated that there were statistically significant differences in depression and dedication scores between the male variable of having children (*p* = 0.000). The depression and dedication scores of male individuals with children were higher than those of individuals without children. Conversely, no statistically significant differences were observed in anxiety (*p* = 0.824), challenge (*p* = 0.967), and control factor (*p* = 0.827) scores.

A statistically significant difference was identified in depression and challenge scores between the female variable of having children (*p* = 0.000). The depression and challenge scores of female individuals who had children were higher than those of individuals who did not have children. Conversely, no statistically significant differences were observed in anxiety (*p* = 0.199), dedication (*p* = 0.553), and control factor (*p* = 0.452) scores. The results are presented in Table 6.

The results of the ANOVA test indicated that there was no statistically significant difference between the scores for anxiety (*p* = 0.107), depression (*p* = 0.305), challenge (*p* = 0.288), dedication (*p* = 0.454), and control factor (*p* = 0.447) across the education level variable (Table 7).

A statistically significant difference was identified in anxiety scores between professions variables (*p* = 0.002). The results of the multiple comparisons Tukey-B test indicate that the anxiety scores of nurses, midwives, and health officers were higher than those of other occupational groups. Significant statistical differences were identified in the challenge (*p* = 0.028) and dedication scores of the professions variable (*p* = 0.009). The challenge and dedication scores of cleaning officers were higher than other occupational groups. No significant differences were observed in depression (*p* = 0.488) and control (*p* = 0.224) scores for the professions variable. The results are presented in Table 8.

There were statistically significant differences in anxiety and depression scores between the working period variables (*p* = 0.000). The Tukey-B multiple comparison test revealed that the anxiety scores of the recently hired personnel were higher than those of the other employees. The depression scores of personnel with a tenure of six to ten years were found to be higher than those of the other groups. The challenge, dedication, and control scores for the tenure variable exhibited statistically significant differences (*p* = 0.121, *p* = 0.176, and *p* = 0.708, respectively). The results are presented in Table 9.

The ANOVA test revealed that the challenge (*p* = 0.037) and control (*p* = 0.001) scores of the shift variable exhibited a statistically significant difference. The Tukey-B multiple comparison results indicated that the challenge and control scores of those who experienced four to seven shifts per month were higher than those of the other groups. Conversely, no statistically significant difference was observed in anxiety (*p* = 0.115), depression (*p* = 0.119), and dedication (*p* = 0.100) scores. The results are presented in Table 10.

The results of the ANOVA test indicated a statistically significant difference in anxiety scores between the levels of contact with COVID-19-positive patients (*p* = 0.004). As a consequence of the Tukey-B multiple comparisons, the anxiety scores of those who had contact with COVID-19-positive patients for a period exceeding eight hours exhibited a statistically significant elevation in comparison to the other groups. Conversely, no statistically significant difference was observed in depression (*p* = 0.257), challenge (*p* = 0.542), dedication (*p* = 0.095), and control factor (*p* = 0.615) scores in relation to contact with COVID-19-positive patients. The results are presented in Table 11.

The normality WIF and tolerance values from the a priori analyses indicate that the assumptions of linearity, multicollinearity, and covariance were not violated. Independent variables were entered in the first step and these variables explained 0.13% of the variance in perceived anxiety, 0.159% of the variance in depression, 0.37% of the variance in challenge, 0.60% of the variance in dedication, and 0.72% of the variance in devotional control. Age affects anxiety; age, education status, and having a child affect depression. The variables of marital status and having a child affect the challenge subdimension of psychological resilience; age, marital status, having a child, professions, professional experience, and work shifts status affect the dedication subdimension; and, finally, age, professions, and professional experience affect the control subdimension. Beta values show that age has the greatest effect on all dependent variables. The results are presented in Table 12 for reference purposes.

The Cronbach alpha reliability values used for the research data are presented in Table 13. The Cronbach alpha values of the scales and their subdimensions were above 0.80. These values indicate a high level of reliability.

## 4. Discussion

In this study, it was observed that female health workers received higher anxiety scores than male health workers; nurses, midwives, and health officers higher than other health workers; health workers with less professional experience higher than health workers with more professional experience; those who were exposed to COVID-19-positive patients for 8 h or more in a working day received higher anxiety scores than those who were exposed for less time. In the analysis of depression scores, it was observed that single female participants had higher depression scores than married female participants, those who had children had higher depression scores than those who did not have children, those with 6–10 years of professional experience had higher depression scores than those with less or more professional experience, and the depression scores of healthcare professionals aged 56 years and over were lower than those of younger healthcare professionals. In other words, young and middle adulthood age groups reported more depressive symptoms than older age groups. The analysis of the anxiety and depression scores of the groups is important not only statistically but also in terms of health. From a clinical point of view, the difference between the groups is particularly important in terms of maintaining or threatening mental health, and the literature review supports these findings.

There are studies in the literature that support the results obtained from anxiety and depression scores. In a study involving 18,171 adult participants, it was reported that 26.6% of the participants had depression symptoms and 28.2% had anxiety symptoms [37]. In a study conducted in Spain with 3480 adult participants, 18.7% of the sample reported depressive symptoms, 21.6% reported anxiety symptoms, and 15.8% reported PTSD symptoms. In the same study, it was reported that female gender and being alone played a significant role in the increase in all symptoms [38]. In another study conducted with 1210 adult participants, it was reported that 16.5% of the participants had depressive symptoms and 28.8% had anxiety symptoms [39]. It is stated that the pandemic outbreak causes negative emotional reactions such as anxiety, fear, and anger in people, increases their stress levels, increases their susceptibility to psychological disorders such as anxiety disorders and depression, and, especially, health workers who fight the epidemic in the field have symptoms related to anxiety and depressive disorders [18]. In the mentioned studies, the participants were not health workers.

However, the results of research involving healthcare workers are similar. In a study of 1257 healthcare workers, it was reported that there was an increase in depression and anxiety scores, especially in female healthcare workers, nurses, and healthcare workers who provide direct diagnosis, treatment, or nursing care to patients with suspected or confirmed COVID-19 [40]. The finding that anxiety and depression are more common in young people in the pandemic is consistent with the literature. Nwachukwu and colleagues reported that stress, anxiety, and depression were found to be more common in young people in their study with 8267 participants in Canada [41]; Wathelet and colleagues reported that in their study involving 69,054 university students during the quarantine process in France (average age was 20 years), high rates of mental health problems were observed in the sample and these were stress, severe depression, and high levels of anxiety, respectively, and the female gender reported more symptoms than males [42]. In a study conducted by Kowal et al. with 53,524 participants from six countries, high levels of stress and anxiety were reported to be associated with young age, being female, and being single [43]. A study conducted in Austria involving 1005 adults similarly reported that 21% of the participants had depressive symptoms and 19% had anxiety symptoms during COVID-19, and that these findings were higher compared to previous epidemiological data.

The same study reported that the COVID-19 pandemic was more symptomatic, especially for young adults (<35 years) and women [44]. In addition, other studies also provide evidence of increased levels of anxiety and depression during the pandemic and that being female, young age, and being single (being alone) may be a risk factor [44,45,46,47]. In the study, the fact that healthcare workers who had children had higher depression scores than those who did not can be explained by the social restrictions brought by the pandemic period (such as curfews, social isolation rules, the obligation of children to be outside the home and under parental supervision only during certain hours) and the transition of education life to the online process, creating a burden on healthcare workers to protect work and family life. In addition, fears of carrying viruses home from work and difficulties with childcare (parents working and having to stay at home because the child cannot go to school) may have contributed to the increase in depressive scores.

In the psychological resilience analyses, it was observed that single female participants scored higher than married female participants, female participants with children scored higher than female participants without children, and cleaning personnel scored higher than those working in other occupations in the challenge and dedication subdimensions of the psychological resilience variable. In addition, it was found that healthcare workers who worked 4–7 shifts in a month scored higher on the challenge and control subdimensions than healthcare workers who worked fewer shifts. The results can be interpreted as follows.

Psychological resilience is a personality trait that reduces the negative effects of stress and prevents organismic tension leading to disease, a cognitive process that facilitates adaptation to life in the face of physical and psychological challenges and causes individuals to gain a positive perspective on themselves in stressful life events [48]. In this context, the stressful nature of the pandemic on healthcare workers (intense workload, risk of being infected, concerns about carrying the infection to the family, loss of infected patients and colleagues, efforts to balance family and social fault with the social constraints brought by the pandemic process, physical difficulties of working in protective equipment, etc.) caused physical and psychological difficulties for healthcare workers. In this study, having children seems to have increased psychological resilience in the subdimensions of dedication and commitment, and the high number of monthly shifts increased psychological resilience in the dimensions of challenge and control.

According to Kobasa, who defines psychological resilience as an individual’s tendency to be interested in various areas of life, dedication, which is a subtype of psychological resilience, is dedication to one’s social environment (including work and family), interpersonal relationships, and one’s own beliefs and values, and this creates a source of strength necessary for the individual to cope with stressful life situations [48]. Sinclair, on the other hand, expresses the trait of dedication by defining the characteristic features of individuals with high level of dedication. According to the theory, individuals with high levels of self-dedication think of themselves and their environment as interesting and worth spending time on. They can find something meaningful and satisfying their curiosity in everything [49].

In the light of this information, it is understandable that healthcare professionals who have children exhibit a higher level of psychological resilience with their dedication and challenge scores. Having children may have enabled the parents to dedicate themselves to the family environment and to cope more with the stressful life situations brought about by the pandemic process (such as care and responsibilities of children, meeting the social and educational needs of children, attempts to protect their children psychologically and socially from the stressful nature of the pandemic process).

The high number of monthly shifts caused an increase in the challenge and control dimensions of psychological resilience. This increase can be understood when considered within the framework of the following information in the literature. In the literature, control is defined as an individual’s belief that he/she can influence the outcomes of events instead of being helpless in the face of events when faced with difficulties in life. The characteristic features of these individuals are self-discipline, success orientation, autonomy, intrinsic motivation, effective use of decision-making skills, and preference for personal freedom and choice [50]. With the increase in the number of shifts, healthcare professionals began to work the night shift (working hours from 16:00 to 08:00 the next day) instead of the day shift. The fact that the number of patients served at night is relatively less than during the day partially alleviates the workload. Reduced workload may have increased the tendency of health workers to take calmer and cooler approaches, to manage personal and professional experience in a less stressful process, to make more self-disciplined and personal decisions.

In challenge, change is perceived as a natural part of daily life. Change is not seen as a threat to security. On the contrary, it is recognized as a stimulus for development. The individual believes that he/she can reorganize the events he/she experiences [51]. Likewise, the challenge scores may have increased for individuals who work more shifts at night, both because they are away from the stressful structure of daytime work and because they have the opportunity to reconsider themselves and events with less workload.

### Limitations

This study has various limitations in different areas. First of all, from the sampling point of view, the data of the study were collected from two hospitals considered as pandemic hospitals that were commissioned to serve only COVID-19 patients, and all other activities were stopped by the Ministry of Health (a pandemic hospital is an inpatient health institution specially commissioned and organized for the treatment of those who carry the disease causing the pandemic. In these hospitals, the aim is to be prepared for the pandemic with all the resources of the health system). For this reason, data were not collected from hospitals that admit COVID-19 patients but do not operate as pandemic hospitals. This situation constitutes a limitation in terms of generalizing the results obtained. In future studies, it is recommended for researchers to collect data simultaneously from healthcare professionals working in hospitals where pandemic cases are admitted other than pandemic hospitals in order to make comparisons and generalizations.

In this study, it was seen that the demographic characteristics of the participants were concentrated in some categories. The fact that the number of female participants was higher than the number of male participants and the number of nurses, midwives, and health officers was higher than the number of physicians is one of the factors that make it difficult to generalize. In future studies, it is recommended that the number of participants should be similar in order to make comparisons and generalizations.

Another limitation of the study is related to the data collection tools. All measurement tools used in the study were self-report scales. It was assumed that the participants answered honestly in these self-assessment tools. In addition, data collection was carried out online due to the pandemic. These reasons constitute a limitation. In addition to face-to-face data collection, it is recommended to use other assessment tools (clinical examination, test, observation, follow-up) in addition to self-report tools in future studies.

Another limitation of this study is that it was cross-sectional. Long-term effects can be analyzed with longitudinal studies.

Another limitation of this study is that it did not adjust for the possible effect of the severity of the pandemic. The study was conducted during a pandemic wave. Therefore, the subjects’ responses may have been influenced by this situation. It is recommended that future studies make comparisons with a variable representing the status or severity of the pandemic, such as ANCOVA models.

Another limitation of this study is that it was conducted using a convenience sampling method; therefore, the results may lead to bias and may not represent the population.

## 5. Conclusions

This study was conducted to examine the psychological effects of the pandemic on healthcare workers during the pandemic period. The data obtained showed that anxiety symptoms were more common in women, nurses, midwives, health officers, those with less professional experience, and those who had long hours of contact with COVID19-positive patients. Depression symptoms were more common in those who were single, had children, and had professional experience of 6–10 years. Being single, having children, and having a certain number of shifts per month (4–7 shifts) had an effect on psychological resilience.

The health sector is one of the most important areas of the service sector. Since the services it provides are related to human health and life, it differs significantly from other institutions operating in this field. Since the work of healthcare professionals is related to the protection, rescue, and sustainability of a person’s life, their work environment is extremely stressful in nature. Considering this situation, their work imposes very heavy responsibilities on them in terms of psychological, social, mental, and labor relations. In addition to these, by prioritizing the physical and mental health of health workers who work on the front line in social traumas such as epidemics, disasters, and wars, the main goal of protecting public health can be achieved. The most recent example of an epidemic is the COVİD-19 pandemic. In such a pandemic that may occur in the future, informing healthcare workers about how to protect themselves from danger, providing psychological interventions, and developing resilience factors will be protective from the development of psychopathology. The involvement of psychologists in health policies created in such situations is important in the biopsychosocial integrity of human beings.

### Suggestions

The health sector is one of the most important service sectors. Because the services it provides are related to human health and life, it differs significantly from other institutions operating in the field. Because the work of health professionals is related to the protection, salvation, and sustainability of human life, their working environment is inherently stressful. In view of this situation, their work imposes very heavy responsibilities on them in terms of psychological, social, spiritual, and working relationships. In addition, the primary goal of protecting public health can be achieved by prioritizing the physical and mental health of health workers who are at the forefront of social traumas such as epidemics, disasters, and wars.

The most recent example of an epidemic is the COVID-19 outbreak. In such a possible future outbreak, informing health professionals about how to protect themselves from hazards, providing psychological interventions and developing resilience factors will protect against the development of psychopathology. In such cases, the involvement of psychologists in health policy is important for the biopsychosocial integrity of the person.

In order to protect mental health, it is recommended that health professionals limit their daily contact with infected patients and reduce long working hours.

It may be protective for health workers to take on more experienced tasks during epidemics, and for those new to the profession to benefit from the experience of experienced health workers and to carry out their tasks under their supervision.

To prevent the workload and professional roles of nurses, midwives, and allied health workers in the epidemic response from leading to burnout, rotating health workers in these positions and increasing the number of workers in this profession may be protective for mental health.

Psychoeducation on anxiety management, rational processing of anxiety, and effective stress management can be provided to health workers, and preventive mental health services can be provided online during pandemic. It is important for healthcare professionals to apply these recommendations at the national level in the decisions to be made by health policymakers and to ensure the mental health of healthcare professionals in the coming pandemic process.

In future studies, it is recommended that researchers simultaneously collect data from healthcare professionals working in pandemic hospitals as well as from other hospitals that receive pandemic cases but are not only pandemic hospitals, and make comparisons and generalizations.

In future studies, it is recommended that the number of participants should be similar in order to make comparisons and generalizations; that data collection methods using assessment tools such as clinical examination, tests, observation, and follow-up should be used to ensure the reliability of the results; and that longitudinal studies should be conducted to obtain data with a high degree of generalizability, in addition to comparisons with results obtained from cross-sectional studies. It is also recommended that future studies use the ANCOVA test to control for covariates between independent groups. Comparison of healthcare professionals working in different services in pandemic hospitals, and pre-test–post-test studies in which sample participants are reassessed after the pandemic are among the recommendations. Finally, it is recommended that future researchers use other sampling methods that are highly representative of the population.

## Figures and Tables

**Table 1:** Demographic characteristics and working conditions of the participants.

Variables		Frequency	Percent
Gender	Male	603	41.02
	Female	837	56.93
	Missing	30	2.04
Age	25 years and below	314	21.36
	26–35 years	349	23.74
	36–45 years	319	21.70
	46–55 years	252	17.14
	56 years and above	206	14.01
	Missing	30	2.04
Marital Status	Single	683	46.46
	Married	757	51.49
	Missing	30	2.04
Having a Child	Yes	719	48.91
	No	721	49.04
	Missing	30	2.04
Education Status	High school graduate	238	16.19
	Associate degree graduate	257	17.48
	University	496	33.74
	Master’s Degree	230	15.64
	MD’s	219	14.89
	Missing	30	2.04
Professions	Physician	281	19.11
	Nurse/Midwife/Health Officer	633	43.06
	Data Entry Staff	263	17.89
	Cleaning Officer	263	17.89
	Missing	30	2.40
Professional Experience	0–5 years	428	29.11
	6–10 years	322	21.91
	11–15 years	307	20.88
	16 years and above	383	26.05
	Missing	30	2.40
Work Shifts Status	Yes	826	56.19
	No	609	41.42
	Missing	30	2.04
COVID-19-Positive Patient Contact Status	Yes	765	52.04
	No	671	45.65
	Missing	34	2.31

**Table 2:** The descriptive statistics of the scales Hospital Anxiety and Depression Scale (HAD) and Psychological Resilience Scale (PRS).

Variable	Min.	Max.	$\bar{X}$	sd	Skewness	Kurtosis
Anxiety	10.00	16.00	11.03	0.38	1.055	1.830
Depression	9.00	13.00	9.95	0.35	1.087	1.577
Challenge	17.00	33.00	25.96	1.12	−1.267	1.167
Dedication	17.00	22.00	17.74	0.49	0.955	1.573
Control	10.00	16.00	10.16	0.89	1.220	1.777

**Table 3:** The having children variable *t*-test results of the Hospital Anxiety and Depression Scale (HAD) and Psychological Resilience Scale (PRS).

	Gender	n	$\bar{X}$	sd	*p*
Anxiety	Female	837	10.46	4.52	0.000
	Male	603	7.20	2.88	
Depression	Female	837	9.24	3.23	0.099
	Male	603	8.61	3.78	
Challenge	Female	837	26.22	5.55	0.991
	Male	603	26.23	5.70	
Dedication	Female	837	16.59	5.47	0.284
	Male	603	17.26	5.76	
Control	Female	837	18.08	3.90	0.167
	Male	603	18.70	4.18	

**Table 4:** The age variable of the ANOVA results of the Hospital Anxiety and Depression Scale (HAD) and Psychological Resilience Scale (PRS).

Variable	Age	n	$\bar{X}$	sd	*p*	Sig. Difference
Anxiety	25 years and below	314	10.99	4.45	**0.000**	1 > 3, 5, 4
	26–35 years	349	10.65	4.50		
	36–45 years	319	9.04	4.93		
	46–55 years	252	7.17	4.17		
	56 years and above	206	8.50	4.96		
Depression	25 years and below	314	8.99	3.01	**0.047**	2 > 1, 3, 4, 5
	26–35 years	349	9.66	3.47		
	36–45 years	319	9.02	3.56		
	46–55 years	252	8.09	3.27		
	56 years and above	206	7.83	2.78		
Challenge	25 years and below	314	26.33	6.00	0.912	-
	26–35 years	349	26.01	5.17		
	36–45 years	319	26.48	5.41		
	46–55 years	252	24.66	6.15		
	56 years and above	206	24.62	6.40		
Dedication	25 years and below	314	16.28	5.81	0.310	-
	26–35 years	349	16.59	5.18		
	36–45 years	319	16.70	5.67		
	46–55 years	252	18.21	5.67		
	56 years and above	206	17.83	5.54		
Control	25 years and below	314	17.90	4.06	0.711	-
	26–35 years	349	18.51	3.93		
	36–45 years	319	18.32	4.02		
	46–55 years	252	18.05	3.96		
	56 years and above	206	17.16	2.63		

- Indicates that the results of multiple comparisons are not significant.

**Table 5:** The marital status variable of The *t*-test results of the Hospital Anxiety and Depression Scale (HAD) and Psychological Resilience Scale (PRS).

Gender	Variable	Marital Status	n	$\bar{X}$	sd	*p*
Male	Anxiety	Single	329	11.0699	0.56685	0.888
		Married	274	11.0766	0.60338	
	Depression	Single	329	9.8298	0.40751	0.426
		Married	274	9.8577	0.45068	
	Challenge	Single	329	26.0729	0.80063	0.586
		Married	274	26.1095	0.84454	
	Dedication	Single	329	17.4498	0.49824	**0.000**
		Married	274	17.2993	0.45878	
	Control	Single	329	10.0365	0.32927	0.698
		Married	274	10.0474	0.36506	
Female	Anxiety	Single	172	11.0174	0.22875	0.141
		Married	665	11.0030	0.05480	
	Depression	Single	172	10.1279	0.42705	**0.000**
		Married	665	10.0211	0.20427	
	Challenge	Single	172	25.4535	2.44067	**0.000**
		Married	665	25.9789	0.71737	
	Dedication	Single	172	18.0058	0.47753	0.947
		Married	665	18.0075	0.22947	
	Control	Single	172	10.3314	1.27529	0.363
		Married	665	10.2436	1.08560	

**Table 6:** The marital status variable of the *t*-test results of the Hospital Anxiety and Depression Scale (HAD) and Psychological Resilience Scale (PRS).

Gender	Variable	Having a Child	n	$\bar{X}$	sd	*p*
Male	Anxiety	Yes	364	11.0687	0.58277	0.824
		No	239	11.0795	0.58514	
	Depression	Yes	364	9.9011	0.36530	**0.000**
		No	239	9.7531	0.49551	
	Challenge	Yes	364	26.0907	0.84633	0.967
		No	239	26.0879	0.78096	
	Dedication	Yes	364	17.5165	0.50042	**0.000**
		No	239	17.1757	0.38139	
	Control	Yes	364	10.0440	0.35325	0.827
		No	239	10.0377	0.33470	
Female	Anxiety	Yes	485	11.0103	0.15040	0.199
		No	352	11.0000	0.00000	
	Depression	Yes	485	10.0639	0.33716	**0.008**
		No	352	10.0142	0.11850	
	Challenge	Yes	485	25.7773	1.69342	**0.014**
		No	352	26.0000	0.00000	
	Dedication	Yes	485	18.0124	0.39082	0.553
		No	352	18.0000	0.00000	
	Control	Yes	485	10.2866	1.18458	0.452
		No	352	10.2273	1.04298	

**Table 7:** The education variable of the ANOVA results of the Hospital Anxiety and Depression Scale (HAD) and Psychological Resilience Scale (PRS).

Variable	Education Status	n	$\bar{X}$	sd	*p*
Anxiety	High school graduate	238	8.75	4.43	0.107
	Associate degree graduate	257	9.83	4.49	
	University	496	10.15	4.52	
	Master’s Degree	230	9.73	4.07	
	MDs	219	7.57	4.63	
Depression	High school graduate	238	8.64	3.06	0.305
	Associate degree graduate	257	9.61	3.53	
	University	496	9.11	3.50	
	Master’s Degree	230	9.40	3.26	
	MDs	219	7.84	3.11	
Challenge	High school graduate	238	26.28	7.11	0.288
	Associate degree graduate	257	26.43	5.87	
	University	496	26.32	5.91	
	Master’s Degree	230	24.13	6.25	
	MDs	219	27.21	6.49	
Dedication	High school graduate	238	17.55	5.82	0.454
	Associate degree graduate	257	16.54	5.25	
	University	496	16.78	5.63	
	Master’s Degree	230	15.23	5.67	
	MDs	219	17.68	5.41	
Control	High school graduate	238	18.57	4.35	0.447
	Associate degree graduate	257	17.92	5.37	
	University	496	18.35	3.85	
	Master’s Degree	230	18.52	3.96	
	MDs	219	18.23	2.34	

**Table 8:** The professions variable of the ANOVA results of the Hospital Anxiety and Depression Scale (HAD) and Psychological Resilience Scale (PRS).

Variable	Professions	n	$\bar{X}$	sd	*p*	Sig. Difference
Anxiety	Physician	281	7.90	4.64	**0.002**	2 > 3, 1, 4
	Nurse/Midwife/Health Officer	633	10.18	4.69		
	Data Entry Staff	263	8.15	3.89		
	Cleaning Officer	263	6.61	4.53		
Depression	Physician	281	8.87	4.56	0.488	-
	Nurse/Midwife/Health Officer	633	9.12	3.28		
	Data Entry Staff	263	10.07	3.25		
	Cleaning Officer	263	8.07	2.69		
Challenge	Physician	281	23.83	5.02	**0.028**	4 > 3, 2, 1
	Nurse/Midwife/Health Officer	633	26.28	5.53		
	Data Entry Staff	263	27.84	5.14		
	Cleaning Officer	263	28.53	7.17		
Dedication	Physician	281	14.83	4.50	**0.009**	4 > 3, 2, 1
	Nurse/Midwife/Health Officer	633	16.72	5.60		
	Data Entry Staff	263	18.07	3.52		
	Cleaning Officer	263	20.84	5.44		
Control	Physician	281	17.41	2.95	0.224	-
	Nurse/Midwife/Health Officer	633	18.22	4.06		
	Data Entry Staff	263	18.53	2.29		
	Cleaning Officer	263	18.23	4.42		

- Indicates that the results of multiple comparisons are not significant.

**Table 9:** The working period variable of the ANOVA results of the Hospital Anxiety and Depression Scale (HAD) and Psychological Resilience Scale (PRS).

Variable	Working Period	n	$\bar{X}$	sd	*p*	Sig. Difference
Anxiety	0–5 years	428	11.00	4.35	**0.000**	1 > 2, 4, 3
	6–10 years	322	10.92	4.57		
	11–15 years	307	7.67	4.07		
	16 years and above	383	8.68	4.99		
Depression	0–5 years	428	9.17	3.06	**0.001**	2 > 1, 3, 4
	6–10 years	322	10.42	3.56		
	11–15 years	307	8.62	3.33		
	16 years and above	383	8.50	3.37		
Challenge	0–5 years	428	26.17	6.00	0.121	-
	6–10 years	322	25.44	4.73		
	11–15 years	307	27.75	4.57		
	16 years and above	383	26.06	5.73		
Dedication	0–5 years	428	16.64	5.61	0.176	
	6–10 years	322	15.72	5.13		
	11–15 years	307	17.82	4.96		
	16 years and above	383	16.99	5.84		
Control	0–5 years	428	18.13	4.06	0.708	-
	6–10 years	322	18.29	4.10		
	11–15 years	307	18.78	3.44		
	16 years and above	383	18.09	4.02		

- Indicates that the results of multiple comparisons are not significant.

**Table 10:** The seizure variable of the ANOVA results of the Hospital Anxiety and Depression Scale (HAD) and Psychological Resilience Scale (PRS).

Variable	Number Shifts in a Month	n	$\bar{X}$	sd	*p*	Sig. Difference
Anxiety	1–3 Shifts	130	9.80	4.53	0.115	-
	4–7 Shifts	397	9.61	4.86		
	More than 7 Shifts	799	10.78	4.72		
Depression	1–3 Shifts	130	9.70	4.54	0.119	-
	4–7 Shifts	397	8.68	3.01		
	More than 7 Shifts	799	9.49	3.38		
Challenge	1–3 Shifts	130	25.36	5.71	**0.037**	2 > 1, 3
	4–7 Shifts	397	27.47	5.42		
	More than 7 Shifts	799	25.79	5.72		
Dedication	1–3 Shifts	130	16.36	6.12	0.100	-
	4–7 Shifts	397	17.75	5.53		
	More than 7 Shifts	799	16.27	5.61		
Control	1–3 Shifts	130	18.46	3.48	**0.001**	2 > 1, 3
	4–7 Shifts	397	19.56	3.96		
	More than 7 Shifts	799	17.77	4.04		

- Indicates that the results of multiple comparisons are not significant.

**Table 11:** The patient contact variable of the ANOVA results of the Hospital Anxiety and Depression Scale (HAD) and Psychological Resilience Scale (PRS).

Variable	Contact Time in One Day	n	$\bar{X}$	sd	*p*	Sig. Difference
Anxiety	Less than 1 h	213	9.98	4.87	0.004	3 > 1, 2
	1–8 h	532	9.06	4.85		
	More than 8 h	520	11.37	4.81		
Depression	Less than 1 h	213	8.83	3.17	0.257	-
	1–8 h	532	9.43	3.76		
	More than 8 h	520	9.98	3.67		
Challenge	Less than 1 h	213	26.68	5.94	0.542	-
	1–8 h	532	25.88	5.76		
	More than 8 h	520	25.66	6.07		
Dedication	Less than 1 h	213	17.14	5.12	0.095	-
	1–8 h	532	17.00	5.98		
	More than 8 h	520	15.52	5.46		
Control	Less than 1 h	213	18.32	4.00	0.615	-
	1–8 h	532	18.24	3.77		
	More than 8 h	520	17.76	4.62		

- Indicates that the results of multiple comparisons are not significant.

**Table 12:** The results of the regression of age, marital status, having a child, educational status, position/title, work experience, and work shifts status on the Hospital Anxiety and Depression Scale (HAD) and Psychological Resilience Scale (PRS).

Variable		B	SE	Beta	t	Sig.	Tolerance	VIF
1	(Constant) ^a^	11.144	0.056		197.538	0.000		
	Age	−0.050	0.024	−0.172	−2.088	**0.037**	0.171	1.883
	Marital Status	−0.022	0.024	−0.026	−0.913	0.362	0.826	1.211
	Having a Child	−0.011	0.021	−0.013	−0.502	0.616	0.970	1.042
	Education Status	0.014	0.032	0.045	0.429	0.668	0.063	1.836
	Professions	0.019	0.032	0.049	0.590	0.555	0.150	1.957
	Professional Experience	−0.017	0.030	−0.051	−0.564	0.573	0.086	1.681
	Work Shifts Status	0.033	0.046	0.041	0.703	0.482	0.198	1.042
a. Dependent Variable: Anxiety: R^2^ = 0.013, F = 2.664, *p* = 0.000								
**1**	(Constant) ^b^	9.786	0.048		203.946	**0.000**		
	Age	0.059	0.020	0.222	2.908	**0.004**	0.101	1.883
	Marital Status	−0.003	0.020	−0.004	−0.158	0.874	0.826	1.211
	Having a Child	−0.079	0.018	−0.108	−4.377	**0.000**	0.960	1.042
	Education Status	0.089	0.027	0.314	3.255	**0.001**	0.063	1.836
	Professions	0.012	0.028	0.033	0.430	0.667	0.100	1.957
	Professional Experience	−0.044	0.025	−0.144	−1.744	0.081	0.086	1.681
	Work Shifts Status	−0.051	0.039	−0.070	−1.284	0.199	0.168	1.042
b. Dependent Variable: Depression: R^2^ = 0.159, F = 38.714, *p* = 0.000								
1	(Constant) ^c^	25.790	0.161		159.962	**0.000**		
	Age	0.009	0.068	0.011	0.134	0.894	0.191	1.883
	Marital Status	0.274	0.067	0.116	4.074	**0.000**	0.876	1.211
	Having a Child	0.160	0.060	0.070	2.647	**0.008**	0.960	1.042
	Education Status	−0.106	0.092	−0.120	−1.159	0.247	0.003	1.836
	Professions	−0.079	0.093	−0.069	−0.848	0.396	0.400	1.957
	Professional Experience	0.031	0.085	0.032	0.364	0.716	0.096	1.681
	Work Shifts Status	−0.078	0.132	−0.034	−0.589	0.556	0.198	1.942
c. Dependent Variable: Challenge: R2 = 0.37, F = 300.333, *p* = 0.000								
1	(Constant) ^d^	17.176	0.046		374.843	**0.000**		
	Age	0.353	0.019	0.961	18.272	**0.000**	0.161	1.883
	Marital Status	0.049	0.019	0.047	2.541	**0.011**	0.826	1.211
	Having a Child	−0.087	0.017	−0.086	−5.044	**0.000**	0.960	1.942
	Education Status	−0.032	0.026	−0.082	−1.231	0.219	0.073	1.536
	Professions	−0.182	0.026	−0.365	−6.919	**0.000**	0.100	1.957
	Professional Experience	0.162	0.024	0.384	6.726	**0.000**	0.086	1.581
	Work Shifts Status	−0.235	0.038	−0.235	−6.253	**0.000**	0.198	1.042
d. Dependent Variable: Dedication: R2 = 0.60, F = 300.333, *p* = 0.000								
1	(Constant) ^e^	9.996	0.126		79.437	**0.000**		
	Age	−0.294	0.053	−0.443	−5.543	**0.000**	0.101	1.883
	Marital Status	−0.093	0.053	−0.049	−1.766	0.078	0.826	1.211
	Having a Child	−0.045	0.047	−0.025	−0.949	0.343	0.960	1.946
	Education Status	0.056	0.071	0.079	0.785	0.433	0.063	1.836
	Professions	0.359	0.072	0.398	4.957	**0.000**	0.110	1.957
	Professional Experience	0.186	0.066	0.245	2.816	**0.005**	0.086	1.681
	Work Shifts Status	−0.128	0.103	−0.071	−1.242	0.214	0.198	1.046
e. Dependent Variable: Control: R2 = 0.72, F = 15.966, *p* = 0.000								

**Table 13:** CronbachAlpha Reability Analysis Results for Scale Scores.

Scales	Cronbach Alfa
Hospital Andety and Depression Scale	0.86
Hospital Anxiety and Depression—Anxiety sub-dimension	0.87
Hospital Andety and Depression Scale—Depression sub-dimension	0.90
Psychological Resilience Scale	0.90
Psychological Resilience Scale—Challenge sub-dimension	0.85
Psychological Resilience Scale—Dedication sub-dimension	0.81
Psychological Resilience Scale—Control sub-dimension	0.84

## Data Availability

The data presented in this study are available to all researchers.
